# Children's active play: self-reported motivators, barriers and facilitators

**DOI:** 10.1186/1471-2458-11-461

**Published:** 2011-06-10

**Authors:** Rowan Brockman, Russell Jago, Kenneth R Fox

**Affiliations:** 1 Centre for Exercise, Nutrition & Health Sciences, School for Policy Studies, University of Bristol, Bristol, UK

## Abstract

**Background:**

Physical activity has important benefits for children's physical health and mental wellbeing, but many children do not meet recommended levels. Research suggests that active play has the potential to make a valuable contribution to children's overall physical activity, whilst providing additional cognitive, social and emotional benefits. However, relatively little is known about the determinants of UK children's active play. Understanding these factors provides the critical first step in developing interventions to increase children's active play, and therefore overall physical activity.

**Methods:**

Eleven focus groups were conducted with 77, 10-11 year old children from four primary schools in Bristol, UK. Focus groups examined: (i) factors which motivate children to take part in active play; (ii) factors which limit children's active play and (iii) factors which facilitate children's active play. All focus groups were audio-taped and transcribed verbatim. Data were analysed using a thematic approach.

**Results:**

Children were motivated to engage in active play because they perceived it to be enjoyable, to prevent boredom, to have physical and mental health benefits and to provide freedom from adult control, rules and structure. However, children's active play was constrained by a number of factors, including rainy weather and fear of groups of teenagers in their play spaces. Some features of the physical environment facilitated children's active play, including the presence of green spaces and cul-de-sacs in the neighbourhood. Additionally, children's use of mobile phones when playing away from home was reported to help to alleviate parents' safety fears, and therefore assist children's active play.

**Conclusions:**

Children express a range of motivational and environmental factors that constrain and facilitate their active play. Consideration of these factors should improve effectiveness of interventions designed to increase active play.

## Background

Regular physical activity in children is associated with numerous benefits for long-term physical health, including lower body mass, blood pressure and insulin levels, and also with improved mental wellbeing [1-4]. Despite these benefits, many children do not engage in the recommended one hour of physical activity on most days of the week [5,6]. Moreover, physical activity levels decline through childhood and adolescence, with the end of primary school (10-11 years) being a critical period of change [7]. As such, there is a need for interventions to increase physical activity among children at the end of primary school. An understanding of the factors that influence children's physical activity, at this age, is therefore an essential first phase in intervention design.

Children typically perform physical activity in several contexts [6]. These include structured activities (e.g. Physical Education (PE) at school and organised sports teams) as well as less structured activities (e.g. walking and cycling to school and 'active play') [8]. Active play has been defined as unstructured physical activity which takes place outdoors in a child's free time [9]. In addition to physical health benefits, active play adds unique contributions to children's development which may not be obtained from more structured forms of physical activity, including creativity, resolving conflicts and informal social engagement away from the influence of adults [10,11].

Whilst active play has been suggested as a potential mechanism for increasing levels of physical activity in this age group [12,13], we know little about how this may be achieved [14,15]. Understanding the determinants of children's active play at the end of primary school (10-11 years) is critical, not only because children's physical activity levels are known to decline steeply over this stage [16,17] but also because it is the period when parents naturally begin to afford their children increased licence to be independently active [18,19].

Despite the emerging acknowledgement that active play makes as an important contribution to increasing physical activity in children [20-22], contemporary children are reported to play outdoors less frequently than previous generations [23,24]. The cause of this has sometimes been attributed to the marked increase in availability of household electronic media [25], including TV, computers and games consoles, which are believed to 'seduce' children away from other children and the outdoors [26]. There is also growing concern that adults are restricting children's independent access to 'traditional' play spaces, such as local parks and streets [27], which limits children's opportunities to engage in active play and to benefit from the social and emotional challenges that active play provides [28,29]. It has been suggested that 'constrained parental behaviour' [30] is mainly motivated by safety concerns, such as road traffic and stranger danger [23]. Although there is emerging evidence of the extent and types of parental restrictions on children's physical activity [9,19] there is a lack of information about its effect on children's active play [29]. There is also little information about how much children constrain their own active play behaviour in response to their perceptions of safety risk.

Understanding the factors which influence active play, from children's perspectives, is fundamental to the development of interventions and policy strategies aimed at promoting active play, and therefore overall physical activity. In particular, there is a need to establish what motivates children to engage in independent active play, whether children are happy with the licence afforded by their parents, what barriers children perceive, and how, in these times of rapid social, environmental and technological change, they and their families are overcoming these barriers. As there is limited available evidence in this area we employed qualitative methods to address three questions among a sample of 10-11 year old children in the UK: 1) why do children engage in active play?; 2) what factors limit children's active play?; 3) what factors facilitate children's active play?

## Methods

A total of 77, 10-11 year old children (28 males and 49 females) were recruited from four primary schools in Bristol, UK. The schools were recruited to represent the socio-economic diversity of the local area based on the Index of Multiple Deprivation (IMD). The IMD is a UK Government-produced measure of deprivation that includes assessments of income, employment, health and education [31]. The IMD was obtained for the postcode of each school and thus represented a measure of deprivation for the school and not the individual participant. Based on the IMDs for the postcodes of all schools within a 10 mile radius of the University of Bristol, one school was recruited from the highest quartile (High Deprivation school), one from the upper-middle quartile (Middle/high Deprivation school), one from the lower-middle quartile (Low/middle Deprivation school) and one from the lowest quartile (Low Deprivation school).

A 'recruitment' session was held for all Year 6 pupils (10-11 years of age) at each school. During the recruitment session the children were invited to participate in a research study about physical activity and play. The study was approved by the School of Applied Community and Health Studies Ethics Committee at the University of Bristol (ref 016/09) and informed parental consent and child assent were obtained for all participants [32]. All Year 6 children who returned a signed consent form were included in the study. The researcher who carried out data collection in schools had enhanced Criminal Records Bureau (CRB) clearance.

Focus groups were chosen as the method of data collection. Focus groups are an effective method of collecting qualitative data from children as the thoughts and ideas of other members of the group often help participants verbalise their responses in a comfortable, safe and supportive environment [33,34]. Depending on the number of consenting participants, two or three focus groups were held at each school, with a range of 4-8 boys and girls in each group. Participants were randomly selected for the focus group by the first author. Each focus group lasted 30-40 minutes, was conducted by the first author and was digitally recorded. All focus groups took place between January and February 2010.

The focus groups had a semi-structured design with follow-up probes on key topics of interest. Questions were developed by the first author and piloted in a school before being finalised. An initial 'ice-breaker' question, intended to make participants feel more at ease with the focus group format, asked, "what do you enjoy doing in your free time after school?" The main focus group questions focused on three themes: (i) factors which motivate children to take part in active play; (ii) factors which limit children's active play and (iii) factors which facilitate children's active play. Children were provided with a definition of active play, which was "any activity which takes place outdoors in your own free time which isn't organised by an adult." The importance of honest, individual answers was stressed, and participants were reminded that the focus group was not a test, in order to limit response bias. Children were also asked to respect the confidentiality of the information shared in the focus group.

### Analyses

All focus group recordings were digitally recorded, transcribed verbatim, and anonymised. All identifying data was removed from the transcripts. Transcripts will be retained in locked storage units for six years and then destroyed by shredding. Thematic analysis was used to reveal the main themes of the research and was conducted in phases. First, key themes were identified by reading the transcripts line by line and marking the text with codes that described the content of the response [35]. Codes were then entered as 'free nodes' (labels that describe themes) into a newly created database in NVivo (Version 2.01, QSR, Southport UK). Codes were checked by the second and third authors before being put into a hierarchical format using 'tree nodes'. The themes were discussed with the second and third authors, for consistency, before being finalised. Text retrievals were then performed on hierarchical codes and contents were interpreted and summarized into themes.

## Results

Eleven focus groups were conducted with 77 participants from four schools, with the sample being 64% female. There were 15 (19.5%) participants from the high deprivation school, 24 (31.0%) from the middle/high deprivation school, 15 (19.5%) from the low/middle deprivation school and 23 (30.0%) from the low deprivation school.

### Factors motivating children to engage in active play

Participants were asked, "Why do you take part in active play?" Responses were divided into four sub-themes: 1) socialising; 2) preventing boredom; 3) health benefits; and 4) freedom. These themes are discussed below.

#### Socialising

One of the primary motivations reported by participants was the sense of enjoyment they experienced through the social aspect of active play:

*"Because I like I being with my friends" *(Male, low deprivation)

*"Because I like meeting people outside that you wouldn't see normally and playing games with them, that you can't really do in the house" *(Female, low/middle deprivation)

*"I like to play with my next door neighbours outside, play games and things" *(Female, high deprivation)

#### Preventing boredom

Many participants, particularly females, also reported being motivated to engage in active play to prevent boredom. Playing outdoors was often regarded as preferable to engaging in sedentary activities such as TV viewing or playing computer/console games.

*"Not just to be sitting at home not doing anything, just watching the TV - it's something to do" *(Female, low deprivation)

*"It's just, better than watching telly" *(Female, high deprivation)

*"Cos it's boring at home sitting in front of the TV and playing your DS and, stuff like that" *(Female, middle/high deprivation)

#### Health benefits

Additionally, many participants recognised the physical and mental health benefits of active play:

*"To burn off energy" *(Male, middle/high deprivation)

*"To keep fit" *(Male, low deprivation)

*"To keep happy" *(Female, middle/high deprivation)

*"I think it's like more healthy and when I'm healthy it makes me feel good" *(Female, low/middle deprivation)

#### Freedom

Finally, many participants reported that feeling a sense of freedom, or escape, from adult control, rules or structured activities were key motivating factors for taking part in active play.

*"We all want to be able to make sure we can do sometimes what we want - not what adults tell us to do" *(Male, high deprivation)

*"Once you've done your homework, sometimes you just...like to go outside and play and with your friends" *(Female, middle/high deprivation)

*"I like playing stuff that's sort of like freely, so you don't have to play against a rule" *(Male, middle/high deprivation)

### Factors limiting children's active play

Participants' responses to factors limiting their active play were divided into two sub-themes: 1) parental constraints; and 2) children's perceived constraints. These themes are discussed below.

#### Parental constraints

Participants were asked, "Do your parents have rules about your outdoor play?" followed by "What are these rules?" The majority of participants reported that their parents had rules about their active play. Most of these rules seemed to be inspired by social fears, such as strangers and teenagers or neighbourhood fears, such as traffic and the neighbourhood feeling generally unsafe.

*"Sometimes it's really like older boys that, you don't know what they could be carrying [weapon]so my mum says like don't go out, and if you go out just go in our front garden" *(Female, high deprivation)

*"Well my mum says like the normal things like don't talk to strangers and that if I'm going out with my friends like not with her, I'm not allowed out when it starts to get dark" *(Female, low deprivation)

*"Well my mum says don't speak to strangers, stay safe and, if I was going in the dark, which I don't, wear like something bright" *(Male, low deprivation)

When asked, "Does this mean you can't play outdoors as much as you'd like to?" participants generally believed their parents' rules to be fair and perceived them to have little effect on their active play:

*"I don't think it affects active play, but they're good because it stops you getting hurt or going too far or doing something you shouldn't" *(Male, middle/high deprivation)

*"It allows them to know like we're ok and where we are, and it also gives us freedom as well" *(Female, low/middle deprivation)

*"It doesn't really affect me cos they're only like simple rules - it's not like rules at school" *(Male, high deprivation)

#### Children's perceived constraints

Participants were asked, "What other things do you think stop you from playing outdoors more?" with prompts including "traffic, other children, stranger danger or weather". Many female participants were constrained from engaging in active play by the presence of groups of older children in their neighbourhoods, who were perceived as threatening and encroaching on the children's preferred play spaces:

*"I sometimes meet them [friends] round the park cos I don't live far from the park, but loads of teenagers hang out there so we don't very often now" *(Female, low/middle deprivation)

*"Well there's some like, really older girls down my road and they sort of like walk up and hang around by my house so, kind of stops me cos they would like come up and sort of like pick on you, so that's why I don't like go out" *(Female, high deprivation)

*"Sometimes when you go to parks, and you see like older children and they're just like hanging about and not really doing what everyone else is doing, climbing up the slides and stuff and kind of ruining things, you feel a bit, like they're taking over, and awkward" *(Female, middle/high deprivation)

The other major barrier reported to affect both male and female children's participation in active play was rainy weather.

*"Um sometimes the weather because when it's snowing it's quite alright cos you can go up the slopes and that but when it's raining, you like can't go out you just have to stay in and, like wait for the rain to go off or, just do nothing" *(Male, middle/high deprivation)

*"Like the weather sometimes cos if it's raining you don't want to go out" *(Female, middle/high deprivation)

*"I go out if it's like sledging weather or it's really sunny...I just don't like it raining really" *(Male, low deprivation)

### Factors facilitating children's active play

Participants' perceptions of factors which facilitated their active play were divided into two sub-themes: 1) neighbourhood play spaces; and 2) technology. These themes are discussed below.

#### Neighbourhood play spaces

Participants were asked, "Where do you meet your friends to play in your neighbourhood?" For boys from the low deprivation school who lived in cul-de-sacs, these were frequently mentioned as a destination for active play, particularly for informal football games:

*"We got a field just in our cul-de-sac, on the other side of our cul-de-sac and we go in there and play football sometimes" *(Male, low deprivation)

*" We go into my cul-de-sac, or, go up to the field" *(Male, low deprivation)

*"Cos I've got like a cul-de-sac outside, and I play football there" *(Male, low deprivation)

Additionally, easily-accessible green spaces were reported to be regularly used for active play by both male and female participants from all schools:

*"I went to my park just down my road, the new one which just opened" *(Male, low/middle deprivation)

*"We go to the park and fields and hills and stuff like that" *(Female, low deprivation)

*"Where I live we have a park right next door so, we just kind of play around in the park" *(Female, high deprivation)

#### Technology

Discussions of how technology facilitated active play arose from the question, 'Do your parents have rules about your outdoor play?' Many participants, both male and female, suggested that their licence to engage in active play was facilitated by owning and using a mobile phone to keep in touch with their parents whilst away from home:

*"My mum and dad don't mind me being out late, cos I got my phone and everything so if I need anything or I'm, coming home I can just tell them so, I'm fine" *(Female, middle/high deprivation)

*"I can do more stuff because I got a phone now, so they can always call me if they think I'm, lost or something" *(Female, low/middle deprivation)

*"Now I've got a phone they just ring me to come back" *(Male, low deprivation)

## Discussion

The data presented in this study indicate that contemporary UK 10-11 year olds value active play and are motivated to engage in it for several reasons, including socialising, preventing boredom, a desire to feel healthy and for the sense of freedom it provides from adult control, rules and structure. Some of these motivational themes support previous research from the physical activity literature, including a US qualitative study with 10-13 year olds, which found the key motivators for physical activity amongst children to include spending time with friends, gaining health benefits and, for girls, preventing boredom [36]. The fact that participants valued active play for the freedom it provides from adult control is significant, as greater freedom to play outdoors with friends, unsupervised by adults, has been linked to higher levels of physical activity in children of this age group [19]. Findings also support previous research showing that, at this age, friends' social support is known to be important for after school activities, whereas parental support may be less important [37,38].

Most participants reported that their parents had rules surrounding their active play, but, in the majority of cases, these restrictions were perceived by the children to be fair and for their own safety. Female participants reported that their own fears, which mainly related to feeling intimidated by groups of teenagers, were more likely to limit their active play than those of their parents'. These findings are consistent with recent qualitative research with children of this age group in Australia [39] and Canada [40]. They also support recent UK-based research with 8-9 year olds which found children to be less concerned with 'traditional' adult fears, such as traffic and 'stranger danger', then they were with the risk posed by other young people in their outdoor spaces [41].

Participants' perception that groups of teenagers can be threatening and limit play opportunities supports previous active play research conducted in Australia, in which 11-year-old participants perceived groups of teenagers' 'bullying' behaviours as a major reason why they did not visit parks more regularly [39]. Our findings add weight to the notion that provision of suitable places for teenagers in local neighbourhoods to socialise, chat and 'hang out' should be a primary focus of local level policy changes, in order to remove barriers to children's outdoor physical activity [26]. Given that teenagers often perceive themselves to have outgrown traditional publicly provided playgrounds [23], it may be appropriate for the teenagers themselves to be involved in the design and planning of such spaces [41].

The weather was reported by participants to be a barrier to physical activity. Although previous research has found children's physical activity can be influenced by features of the physical environment [14], seasonality and weather conditions have been relatively overlooked in the literature [42]. Contrary to suggestions that winter weather is a barrier to children's outdoor play [43-45], this research found that it was rain that hindered children rather than snowy weather, the latter of which was reported to provide additional opportunities for active play, such as sledging and snowball fights with friends. However, it is important to note that, in contrast to the snowfall experienced by North American and Canadian children, the snowfall in the UK is relatively infrequent, light and short-lived. Nevertheless, the findings reinforce the need to provide seasonal and meteorological data, specifically hours of daylight, temperature and levels of precipitation, when developing interventions to increase children's outdoor physical activity.

Further research is needed to examine how UK children may overcome the barrier of wet weather. Interestingly, a recent survey by the Scottish Parent Teacher Council (SPTC) and the play charity 'Grounds for Learning', found strong parental support for children to be allowed to play outside at school break time in wet weather, as long as they had appropriate clothing [46]. Encouraging schools to permit their pupils to play outdoors during wet break times, perhaps even providing them with free wet weather clothing, could mean that playing in the rain becomes more acceptable to children and may therefore reduce the likelihood of it preventing outdoor play in their leisure time.

Participants reported that some aspects the physical environment positively affected their active play opportunities. For example, children from the low deprivation school who lived in cul-de-sacs reported using these spaces to meet and engage in active play with neighbourhood friends. This corresponds with a qualitative study with Australian parents, who reported that living in a court or cul-de-sac was positively associated with active play in the child's street [47] and that children living in these locations were more likely to play independently and unsupervised by adults, as they are relatively safe from through traffic [9]. Additionally, many participants reported playing in neighbourhood green spaces, which have previously been found to be a common venue for physical activity among young people [48] and have traditionally been recognised as play spaces for children [27,49]. Thus, designing street networks that include cul-de-sacs and providing green spaces that are easily accessible to children may maximize opportunities for children to engage in active play in their neighbourhoods [15].

Mobile phones were reported as being regularly used by children to keep in touch with parents when playing outdoors and that, because of this, their parents allowed them greater independent mobility. Interestingly, recent consumer surveys in the UK have reported that three quarters of all children aged between 7 and 15 years own 'at least' one mobile phone [50] and, also, that the number of parents who believe it is acceptable to allow a child under 12 years to own a mobile phone has increased by more than a third in the last few years [51]. Given that technology is often regarded as a barrier to active play, the notion that possessing and using a mobile phone when playing outdoors might actually increase children's independent mobility and, therefore, opportunities to engage in active play, is an important one and worthy of further investigation.

### Limitations

The direct involvement of children in this study and the ability to gather information from them about their own local neighbourhood was a strength of this piece of research. The validity of the results is, however, dependent on the children's ability to understand the focus group questions and accurately recall past events and experiences. Additionally, the random sampling of participants and mixed gender make-up of groups may have had an impact on how participants responded to focus group questions. However, the researcher made an effort to create an environment that was non-threatening, confidential and stimulated the freedom to talk openly among focus group members.

It is important to acknowledge that the findings of this study cannot be generalised across other populations as they only represent the views of groups of children from four primary schools living in inner-city and suburban areas of Bristol. Additionally, the research was conducted in winter so seasonality may have been a confounding influence on our findings. Also, this study provided an initial examination of the key factors influencing children's active play, and how play may be used as a mechanism for increasing children's physical activity. Further work will be needed to provide greater detail on some of the emergent themes. Finally, although previous work has indicated differences in reported active play behaviour according to gender [52] and level of deprivation [12], further research is needed, using single sex focus groups and greater sample sizes, in order to clarify these associations.

## Conclusions

Contemporary British 10-11 year old children appear to value active play, but may be constrained by certain social and environmental factors. Children seem to be motivated to engage in active play in their leisure time and this reinforces the need to encourage collaborative efforts between local governments, urban planners and community groups to provide children with safe and accessible spaces to play outdoors. Both the built environment and green spaces appear to play a significant role in promoting children's active play. Additionally, increased ownership and use of mobile phones may be an important emerging facilitator. Strategies that build on these factors could form the foundation of approaches to increasing children's active play and therefore overall physical activity, among this important target age group. Further research should quantitatively assess these influences on active play in a larger sample of children, using objective measures.

## Competing interests

The authors declare that they have no competing interests.

## Authors' contributions

The study was designed by RB, RJ and KF. Analysis was performed by RB, RJ and KF. The first draft of the paper was written by RB and all authors provided critical input and revisions. All authors read and approved the final manuscript.

## Pre-publication history

The pre-publication history for this paper can be accessed here:

http://www.biomedcentral.com/1471-2458/11/461/prepub
